# Early Tip Capture Release and Push-Up Technique Using the Valiant Stent Graft System for Aortic Arch Aneurysms

**DOI:** 10.3400/avd.nmt.25-00069

**Published:** 2025-09-11

**Authors:** Shizuyuki Dohi, Yasutaka Yokoyama, Atsumi Oishi, Yuichiro Sato, Daisuke Endo, Yoichiro Machida, Jiyoung Lee, Taira Yamamoto, Akie Shimada, Minoru Tabata

**Affiliations:** 1 Department of Cardiovascular Surgery, Juntendo University Nerima Hospital, Tokyo, Japan; 2 Department of Cardiovascular Surgery, Juntendo University Hospital, Tokyo, Japan; 3 Department of Cardiovascular Surgery, Juntendo University Shizuoka Hospital, Izunokuni, Shizuoka, Japan; 4 Department of Cardiovascular Surgery, Todachuo General Hospital, Toda, Saitama, Japan

**Keywords:** aortic arch aneurysm, thoracic endovascular aneurysm repair, Valiant stent graft system

## Abstract

During thoracic endovascular aneurysm repair for aortic arch aneurysms, deployment of the stent graft parallel to the aortic neck is crucial to preventing a type Ia endoleak from the proximal end. We report the early tip capture release and push-up technique that comprises early release of the proximal bare stent, which is typically deployed last during stent graft deployment, followed by a push-up maneuver after landing the proximal edge, thus allowing conformation to the aortic morphology. This technique is effective even for complex aortic arch anatomy.

## Introduction

During thoracic endovascular aneurysm repair (TEVAR) for aortic arch aneurysms, the prevention of a type Ia endoleak is important to achieving good long-term results. The surgeon is required to place the stent graft accurately and parallel to the aortic neck to ensure a long landing zone without creating a bird-beak configuration. We describe the early tip capture release and push-up technique using the Valiant stent graft system (Medtronic, Santa Rosa, CA, USA) to conform to the 3D morphology of the aorta.

## New Methods

**Fig. 1:**
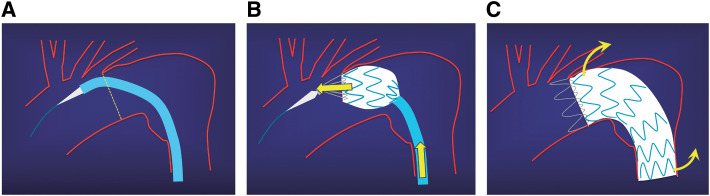
(**A**) When enclosed within the sheath, the Valiant stent graft (Medtronic, Santa Rosa, CA, USA) remains rigid and tends to follow the shortest path; therefore, conforming to the greater curvature of the aortic arch is difficult despite wire manipulation. (**B**) Even when the push-up maneuver is attempted while the proximal tip is still captured, the stent graft is not securely fixed to the aortic wall; it merely shifts proximally. (**C**) Deployment without proper alignment with the greater curvature results in significant spring-back forces at both the proximal and distal ends of the stent graft.

Typically, the Valiant stent graft is fully unsheathed and deployed by releasing the tip capture at the distal end. However, while within the sheath, the Valiant device is relatively rigid and may follow the shortest route, resulting in failure to conform to the aortic outer curvature before deployment. Particularly in cases involving a short neck and large aneurysm on the greater curvature, pushing to achieve conformity during deployment may cause displacement of the proximal tip, resulting in difficulty achieving accurate placement (**Figs. 1A** and **1B**).

Deployment without pushing may result in a bird-beak configuration,^1)^ thus increasing the risk of delayed migration caused by flow dynamics. Additionally, increased spring-back forces at both the proximal and distal ends create higher risks of retrograde type A dissection (RTAD) and distal stent-induced new entry (D-SINE) (**Fig. 1C**).^2)^

**Fig. 2:**
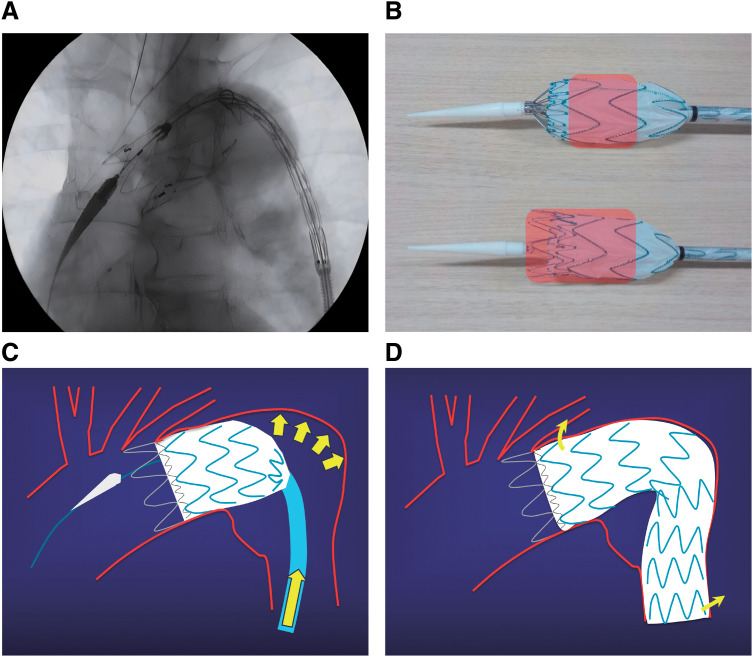
(**A**) After deploying 2 to 2.5 covered stents, the proximal bare stent is released to achieve early proximal landing. (**B**) Releasing the proximal bare stent increases the area of apposition (red), thus enhancing proximal fixation. (**C**) After the proximal segment is landed and secured to the aortic wall, a controlled push-up maneuver allows deployment along the greater curvature. (**D**) Schematic illustration of the final configuration after completion of the push-up maneuver. The spring-back forces applied to both ends of the stent graft are reduced.

To enable the push-up maneuver from this state, the slider should be rotated and 2 to 2.5 covered stents should be deployed; then, the tip capture release handle should be promptly pulled to allow early release of the proximal bare stent and anchoring of the proximal end (**Fig. 2A**). Quick activation of the release handle prevents distal displacement even without rapid pacing.

Landing 3 to 3.5 stents, including the proximal bare stent (**Fig. 2B**), stabilizes the position during the push-up maneuver. After stabilization, the push-up maneuver should be performed to align the graft parallel to the proximal neck and conform to the aortic curvature (**Figs. 2C** and **2D**).

The push-up maneuver causes horizontal folds in the stent graft fabric, which may help prevent type Ia endoleaks. However, excessive pushing can result in graft folding or invagination; therefore, careful deployment guided by tactile feedback and the sensation of blood flow resistance is essential.

**Fig. 3:**
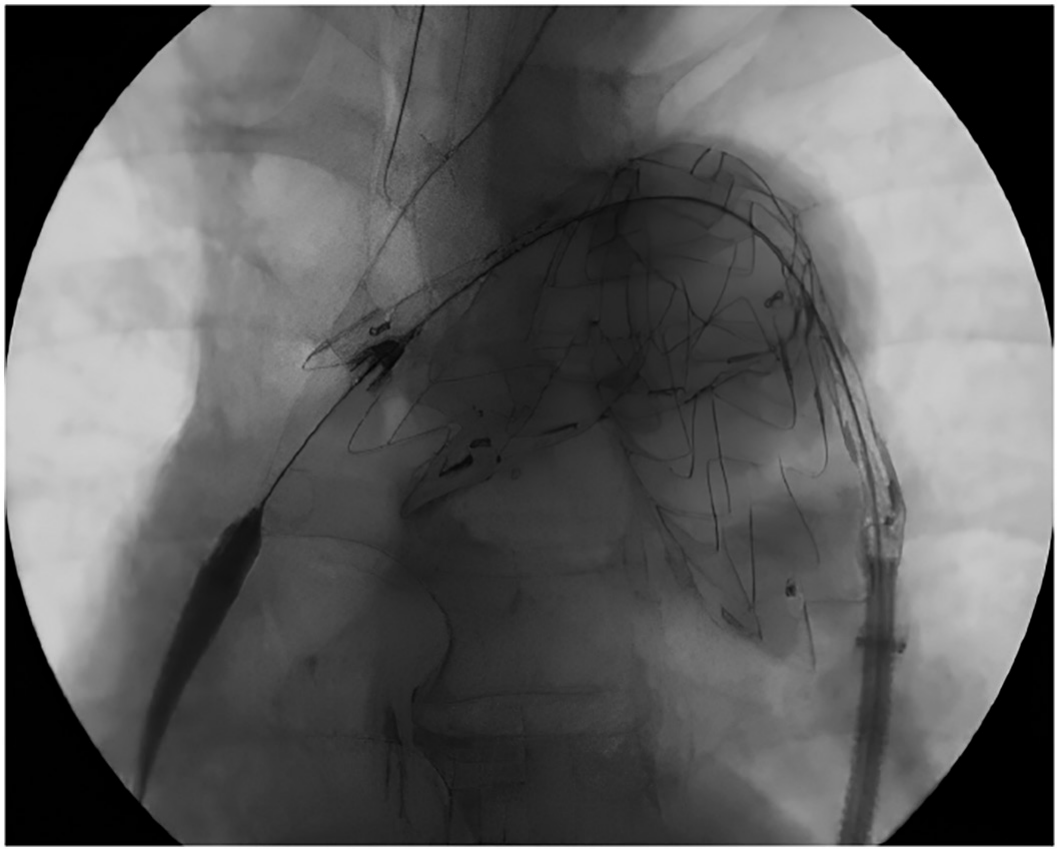
Final fluoroscopic image after completion of deployment with the push-up maneuver. The proximal tip has markedly shifted proximally, thus demonstrating that release of the captured tip enables the stent graft to conform to the aortic curvature in response to the push-up maneuver.

Ultimately, the stent graft conforms well to the aortic curvature, and the proximal tip moves proximally with the force of the pushing maneuver (**Fig. 3**).

## Discussion

Preventing type Ia endoleaks during TEVAR requires sufficient apposition to the proximal neck and deployment resulting in conformation to the aortic anatomy. Compared to its predecessor, the Talent graft (Medtronic), the Valiant graft offers improved conformability and enhanced wall apposition attributable to higher radial force.^3)^ With the Captivia system, a feature specific to the Valiant stent graft, the tip capture mechanism has improved operability, thus providing greater control during complex arch deployment, including the push-up maneuver.^4)^ During the VALOR II trial, the Valiant stent graft was associated with a type Ia endoleak rate of only 13% at 12 months, thus demonstrating high fixation and apposition.^5)^

However, precise deployment remains challenging when cases involve short landing zones or sharp curvatures. The push-up maneuver during deployment is difficult with conventional methods, which increase the risks of bird-beak formation, type Ia endoleaks, RTAD, and D-SINE.^6)^

The early tip capture release and push-up technique enables precise and stable placement by maximizing the conformability of the Valiant stent graft during TEVAR for aortic arch aneurysms. From August 2016 to December 2024, we applied this technique in 52 consecutive cases with complex aortic arch anatomy. In all cases, deployment was successfully completed without intraoperative proximal or distal displacement of the stent graft. No complications, including fabric invagination or malposition, were encountered. Postoperative computed tomography confirmed satisfactory wall apposition and smooth conformation of the stent graft to the curvature of the aortic arch in all cases.

By achieving stable proximal fixation prior to the push-up maneuver, this technique facilitates deployment with conformation to the aortic curvature, reduces spring-back force, and helps prevent complications such as RTAD and D-SINE (**Fig. 2D**).

The formation of horizontal folds in the graft fabric resulting from the push-up maneuver is expected to further prevent type Ia endoleaks, especially in highly curved segments.^7)^ This benefit is particularly notable in cases with short landing zones and severe curvature.

The purpose of this technique is not to force the device into position, but rather to achieve coaxial alignment of the proximal stent graft with the aortic axis followed by conformity of the graft body along the greater curvature of the aorta. The push-up maneuver should be performed with attention to the vector and direction of applied force based on tactile feedback during deployment. If resistance is felt or poor radial expansion of the stent frame is observed under fluoroscopy, then the operator should reduce the pushing force and avoid performing any further push-up maneuver. Because the stent skeleton is visible during deployment, it is possible to detect signs of suboptimal expansion and respond accordingly. Although fabric invagination is theoretically possible if excessive force is applied after proximal release, we have not encountered this complication in our experience. Because the proximal bare stent has already been released and disconnected from the inner sheath, fabric invagination is theoretically possible if excessive force is applied; however, we have not observed this complication in our experience. The Valiant stent graft system is particularly compatible with this technique because its delivery sheath possesses moderate rigidity. This structural property enables the operator’s hand movements to be transmitted directly to the stent graft tip in real time, thus facilitating precise millimeter-level positioning. Such fine control is critical when performing the push-up maneuver. In contrast, the deployment system of the Conformable GORE TAG (C-TAG; W. L. Gore & Associates, Flagstaff, AZ, USA)—including both the earlier version that deploys from the center and the current C-TAG with Active Control System (C-TAG ACS) that deploys from distal to proximal—does not permit manual control of the stent graft during deployment. Therefore, an intentional push-up maneuver based on the operator’s feedback and anatomical alignment is not feasible. This highlights a practical advantage of the Valiant system that makes it particularly well-suited for this technique in terms of operator control and maneuverability within the aortic arch.

This technique does require appropriate anatomical understanding and intraoperative judgment. However, the procedure itself is simple and reproducible. Even less experienced operators can perform it safely, particularly under the guidance of a mentor who is familiar with the procedure. Although rapid pacing is not routinely necessary, it may be considered in cases with more complex anatomical configurations for which hemodynamic stability is critical.

A preoperative imaging assessment is vital to ensuring appropriate case selection. Although the decision to use this technique is based on a comprehensive evaluation of aortic morphology, certain anatomical indicators may serve as practical thresholds for its application. These include a proximal landing zone length ≤20 mm, an aortic arch angle ≤95°, and aneurysmal protrusion along the greater curvature. These features were consistently observed in cases for which this technique was utilized. Additionally, these features are consistent with anatomical predictors associated with type Ia endoleaks in arch TEVAR that have been previously reported.^8)^

Although the early tip capture release deviates from the standard deployment sequence, the release mechanism of the Valiant system is structurally simple and functions reliably provided that the proximal bare stent segment has exited the outer sheath. Through repeated deployment testing, we have confirmed that this maneuver does not result in mechanical failure. Furthermore, once the graft is anchored to the aortic wall before release, it is subject to predictable distal forces caused by blood flow, regardless of the release timing. When performed with an appropriate understanding of device mechanics and aortic anatomy, this technique does not introduce additional procedural risk. Therefore, formal ethical approval was not considered required to report this technical modification.

This technique does not have absolute contraindications provided that the use of the Valiant stent graft itself is not contraindicated. The procedure is a controlled intraoperative modification performed within the standard use of the device. However, it is unlikely to offer added benefits in cases with a long, straight proximal landing zone for which conventional deployment provides adequate wall apposition. In such cases, application of this technique may be unnecessary. Although the maneuver is technically straightforward, we recommend that less experienced operators should perform it under the supervision of clinicians who are familiar with the Valiant system and this technique.

In conclusion, this technique offers a simple and reproducible strategy for improving proximal fixation and arch conformability during TEVAR with the Valiant stent graft. Further studies are needed to determine whether its outcomes are improved compared to those of conventional methods.

## Declarations

### Availability of data and materials

Data sharing is not applicable to this article as no datasets were generated or analyzed during the current study.
